# Frequency-Stable Ionic-Type Hybrid Gate Dielectrics for High Mobility Solution-Processed Metal-Oxide Thin-Film Transistors

**DOI:** 10.3390/ma10060612

**Published:** 2017-06-03

**Authors:** Jae Sang Heo, Seungbeom Choi, Jeong-Wan Jo, Jingu Kang, Ho-Hyun Park, Yong-Hoon Kim, Sung Kyu Park

**Affiliations:** 1 School of Electrical and Electronic Engineering, Chung-Ang University, Seoul 06980, Korea; heojs38@gmail.com (J.S.H.); jzw0108@nate.com (J.-W.J.); uangelion@gmail.com (J.K.); hohyun@cau.ac.kr (H.-H.P.); 2 SKKU Advanced Institute of Nanotechnology (SAINT) and School of Advanced Materials Science and Engineering, Sungkyunkwan University, Suwon 16419, Korea; oyenice@skku.edu

**Keywords:** metal-oxide semiconductors, thin-film transistors, hybrid gate dielectric, low temperature solution-process, high mobility

## Abstract

In this paper, we demonstrate high mobility solution-processed metal-oxide thin-film transistors (TFTs) by using a high-frequency-stable ionic-type hybrid gate dielectric (HGD). The HGD gate dielectric, a blend of sol-gel aluminum oxide (AlO*_x_*) and poly(4-vinylphenol) (PVP), exhibited high dielectric constant (ε~8.15) and high-frequency-stable characteristics (1 MHz). Using the ionic-type HGD as a gate dielectric layer, an minimal electron-double-layer (EDL) can be formed at the gate dielectric/InO*_x_* interface, enhancing the field-effect mobility of the TFTs. Particularly, using the ionic-type HGD gate dielectrics annealed at 350 °C, InO*_x_* TFTs having an average field-effect mobility of 16.1 cm^2^/Vs were achieved (maximum mobility of 24 cm^2^/Vs). Furthermore, the ionic-type HGD gate dielectrics can be processed at a low temperature of 150 °C, which may enable their applications in low-thermal-budget plastic and elastomeric substrates. In addition, we systematically studied the operational stability of the InO*_x_* TFTs using the HGD gate dielectric, and it was observed that the HGD gate dielectric effectively suppressed the negative threshold voltage shift during the negative-illumination-bias stress possibly owing to the recombination of hole carriers injected in the gate dielectric with the negatively charged ionic species in the HGD gate dielectric.

## 1. Introduction

Solution-processed metal-oxide thin-film transistors (TFTs) are emerging as a potential replacement for amorphous and low-temperature polycrystalline silicon TFTs in active-matrix electronics including displays, sensor arrays, and driving circuits due to their relatively high carrier mobility and good scalability over a large area [1,2,3,4]. In addition, their amorphous nature and high optical transparency in the visible range may open up a new promising application for transparent and wearable electronics. In general, the metal-oxide TFTs such as indium-gallium-zinc oxide exhibit a higher carrier mobility than that of the amorphous silicon and organic TFTs [5,6]. However, in order to realize high definition, high frame-rate displays, and the relevant driving circuitry, the carrier mobility must be further improved while exhibiting good operational stability. For these reasons, various metal-oxide semiconductors [7,8], gate dielectrics [9,10], novel device structures [11,12], and post treatments [13,14] have been proposed to enhance the carrier mobility of these devices. Among the various approaches in achieving high mobility metal-oxide TFTs, using an ionic-type gate dielectric is a promising method of achieving both the high mobility and low voltage operation characteristics [15,16,17,18,19]. Previously, ion-gel-type gate dielectrics [20], protonated SiO_2_ gate dielectrics [21], and electrolyte gate dielectrics have been explored. Although these ionic-type gate dielectrics showed promising results for achieving high mobility devices, their limited operation at high frequencies may hinder their practical application in active-matrix electronics. In this respect, new types of ionic-type gate dielectrics, which are capable of operating at high frequencies, are now in high demand. Moreover, for the consistency in the fabrication process of oxide TFTs, solution processing of frequency-stable ionic-type gate dielectrics will be more favorable.

Here, we demonstrate solution-processed ionic-type gate dielectrics based on AlO*_x_* and a hybrid of AlO*_x_* and poly(4-vinylphenol) (PVP) materials. In particularly, the low-temperature solution-processed hybrid gate dielectric (HGD) exhibited reasonably high capacitance and dielectric constant, and only a slight decrease in areal capacitance was observed at high frequencies up to 1 MHz along with minimal hysteresis due to the local confinement of mobile ions inside the polymer networks and consequent suppression of electron-double-layer (EDL) effects [22]. In addition, using the solution-processed HGD gate dielectrics in indium oxide (InO*_x_*) TFTs, relatively high mobilities up to 24 cm^2^/Vs were achieved due to the formation of an acceptable EDL at the gate dielectric/InO*_x_* interface for high frequency applications. These results demonstrate that the low-temperature solution-processed HGD gate dielectrics may enable the high-mobility, low-voltage, and high-frequency operation of oxide TFTs.

## 2. Experimental Procedure

**Figure 1:**
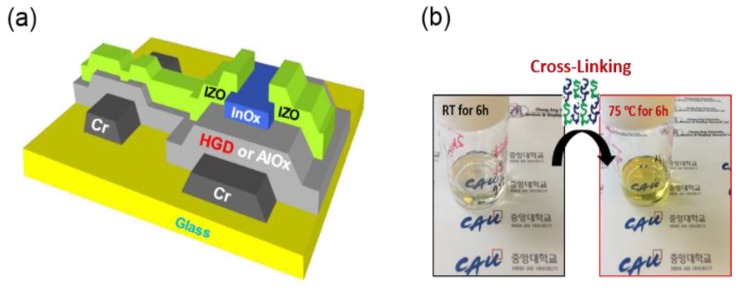
(**a**) Schematic illustration of InO*_x_* TFTs using AlO*_x_* or HGD gate dielectric layer; (**b**) Photographs of an HGD solution before and after a thermal cross-linking.

For the fabrication of an AlO*_x_* gate dielectric, an AlO*_x_* precursor solution was prepared by dissolving 0.8 M aluminum nitrate nonahydrate in 2-methoxyethanol (2-ME) followed by vigorously stirring at 75 °C for 12 h. For the HGD gate dielectric, a poly(4-vinylphenol) (PVP) precursor was dissolved by stirring in 2-ME for 12 h to form a 5 mg mL^−1^ solution. Then, an additional 5% PVP (AlO*_x_*: 5% PVP) was added to the AlO*_x_* precursor solution and stirred for 6 h at 75 °C. For the InO*_x_* channel formation by a solution process, 0.1 M indium nitrate was dissolved in 2-ME and stirred for 12 h at 75 °C. For the electrical characterization of solution-processed gate dielectrics, a heavily-doped n^+^ Si wafer was used as a substrate, and Si wafer/AlO*_x_*(or HGD)/Al (metal-insulator-metal; MIM) and Si wafer/AlO*_x_*(or HGD)/InO*_x_*/Al (metal-insulator-semiconductor; MIS) structures were constructed. The top Al was deposited by using a thermal evaporator with a shadow mask (effective area: 100 × 100 μm^2^). For the fabrication of InO*_x_* TFTs, a borosilicate glass was used as a substrate. Then, Cr gate electrode was deposited by e-beam evaporation and patterned by using standard photolithography and wet etching processes. Afterwards, the AlO*_x_* or HGD precursor solution was spin-coated over the gate electrode to form a gate dielectric layer. Thermal annealing treatment at 150, 250, 350, and 450 °C for 30 min was carried out in an ambient air condition, resulting in thicknesses of 89, 60, 50 and 40 nm, respectively. After the formation of the gate dielectric, an InO*_x_* precursor solution was spin-coated to form a channel layer and thermally annealed at 250 °C for 30 min, resulting in a thickness of 7 nm. For the source/drain electrodes, 60-nm-thick indium zinc oxide (IZO) was deposited by sputtering and patterned by a lift-off process (Figure 1a). The channel width and length of the InO*_x_* TFTs were 100 μm and 10 μm, respectively. The thickness of the gate dielectric layer was measured by using spectroscopic ellipsometry, and to obtain the dielectric constant (ε*_r_*) of the film, the capacitance value (*C*) was first assessed by using a precision LCR meter and the dielectric constant was extracted using the following equation:

$$C=\varepsilon_{0}\varepsilon_{r}\frac{A}{d}$$

where *C* is the capacitance, *A* is the overlapped area between top and bottom electrodes, *d* is the thickness of gate dielectric layer, and ε_0_ is the vacuum permittivity, respectively.

To evaluate the dielectric properties of AlO*_x_* and HGD films, areal capacitance vs. frequency and leakage current density vs. electric field were analyzed using a precision LCR meter and a semiconductor parameter analyzer, respectively. In addition, the electrical characterization and bias stability tests for the InO*_x_* TFTs were carried out using a semiconductor parameter analyzer under an ambient air condition with a relative humidity of ~33% and a temperature of 24 °C. For analyzing the transfer characteristics, the gate voltage was swept from −5 to 6 V with a voltage step of 0.1 V. Additionally, it should be noted that, since the degree of hysteresis in the transfer curves and the field-effect mobility of InO*_x_* TFTs with low-temperature annealed gate dielectric can vary with the gate-voltage sweep rate, we applied a gate-voltage sweep rate of 0.13 V/s for all TFT measurement.

## 3. Results and Discussion

**Figure 2:**
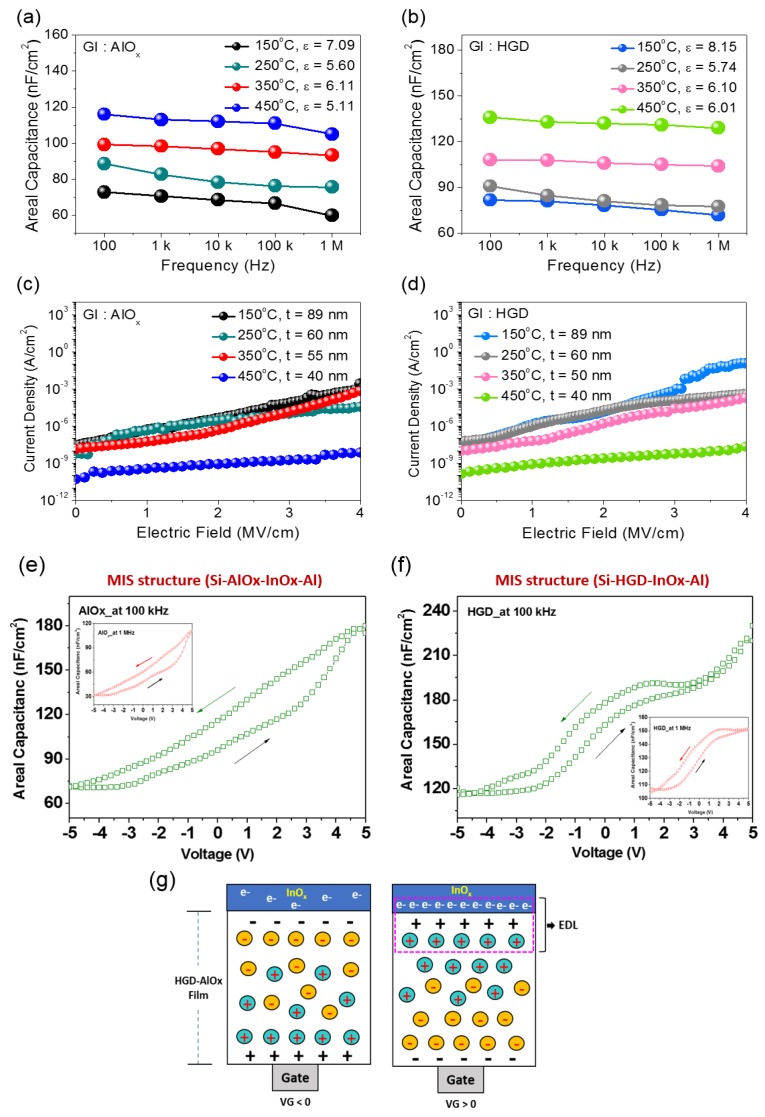
Electrical characteristics for solution-processed AlO*_x_* or HGD dielectric layer using metal-insulator-metal structure (MIM; Si/AlO*_x_* or HGD/Al) with the different annealing conditions (at 150, 250, 350, and 450 °C). The areal capacitance per area-frequency (C-F) of solution-processed (**a**) AlO*_x_* and (**b**) HGD dielectric layer; leakage current density-electric field (J-E) of solution-processed (**c**) AlO*_x_* and (**d**) HGD dielectric layer; the C-V characteristics of an (**e**) AlO*_x_* and (**f**) HGD gate dielectric (150 °C) at 100 kHz and 1 MHz (inset) using metal-insulator-semiconductor structure (MIS; Si/AlO*_x_* or HGD/InO*_x_*/Al); (**g**) the polarization mechanisms and electric double layer formation in InO*_x_* TFTs with HGD dielectric layer.

In order to examine the dielectric properties of solution-processed AlO*_x_* and HGD layers, the areal capacitance vs. frequency (C-F) and the current density vs. electric field (J-E) characteristics were measured. In addition, the difference in the surface morphology of AlO*_x_* and HGD films is negligible. Figure 2a,b show the areal capacitance vs. frequency data for AlO*_x_* and HGD gate dielectrics, respectively. In the case of the AlO*_x_* gate dielectric, the capacitance value and the corresponding dielectric constant showed an increasing trend with the annealing temperature. Particularly, with an annealing temperature of 150 °C, the dielectric constant was 7.09 (thickness of 89 nm), but it decreased to 6.11 and 5.11 when the annealing temperature was increased to 350 °C and 450 °C, respectively. In the case of HGD gate dielectric, a similar behavior was observed as shown in Figure 2b. With an annealing temperature of 150 °C, HGD gate dielectric exhibited a dielectric constant of 8.15, and decreased to 6.10 and 6.01 when the annealing temperature was increased to 350 °C and 450 °C, respectively. It should be noted that the areal capacitance value and the corresponding dielectric constant were higher in the HGD gate dielectric, which can be attributed to the mobile ions in the polymer network combined with AlO*_x_* film. To further investigate the insulating properties, the current density vs. electric field measurements for AlO*_x_* and HGD gate dielectrics were carried out. Figure 2c,d show the current density vs. electric field data for AlO*_x_* and HGD gate dielectrics, respectively. In the case of AlO*_x_* gate dielectric, even a low temperature annealing of 150 °C resulted in reasonable insulating properties. With the annealing temperature of 150 °C, the leakage current density was 4.45 × 10^−6^ A/cm^2^ at an electric field of 2 MV/cm. Further increasing the annealing temperature to 350 °C and 450 °C improved the insulating properties, and the current density was decreased to 4.44 × 10^−7^ A/cm^2^ and 8.29 × 10^−10^ A/cm^2^, respectively. In the case of HGD gate dielectric, even a low temperature annealing of 150 °C resulted in reasonable insulating properties. At an annealing temperature of 150 °C, the leakage current density was 1.47 × 10^−5^ A/cm^2^ at an electric field of 2 MV/cm. Increasing the annealing temperature to 350 °C and 450 °C improved the insulating properties and the current density was decreased to 1.47 × 10^−6^ A/cm^2^ and 2.37 × 10^−9^ A/cm^2^, respectively. Additionally, an AlO*_x_* and HGD film with the annealing temperature of 250 °C have insulator properties similar to those of 150 °C-annealed gate dielectric films, while thickness were decreased from 89 to 60 nm. The slightly high leakage current density observed in HGD gate dielectrics provides that a profound number of mobile ions may exist in the gate dielectric layer. However, even with the mobile ions, the leakage current density is reasonably low to be utilized as a gate dielectric layer.

The presence of mobile ions in the solution-processed AlO*_x_* and HGD gate dielectrics were further validated by measuring the areal capacitance change as a function of bias polarity. Figure 2e,f show the areal capacitance vs. voltage (C-V) plots for AlO*_x_* and HGD gate dielectrics annealed at 150 °C, respectively. Here, the C-V characteristics were obtained using an MIS structure of Si wafer/AlO*_x_*(or HGD)/InO*_x_*/Al at frequencies of 100 kHz and 1 MHz (inset). As displayed, the areal capacitance increases when the bias polarity changed from negative to positive bias, which can be attributed to the formation of an EDL at the AlO*_x_*(or HGD)/InO*_x_* interface. Particularly, in the case of AlO*_x_* gate dielectric, the hysteresis was comparably larger than that of an HGD gate dielectric, possibly indicating a larger amount of residual mobile ions present in the film. As schematically illustrated in Figure 2g, when a negative bias is applied to the Si wafer, positively charged mobile ions accumulate near the AlO*_x_*(or HGD)/Si wafer interface, while the negatively charged mobile ions move toward the AlO*_x_* or HGD/InO*_x_* interface. Since the majority carrier in the InO*_x_* semiconductor is an electron (an n-type), the negatively charged mobile ions near the AlO*_x_*(or HGD)/InO*_x_* interface repel the electrons in the InO*_x_* channel. However, when a positive bias is applied to the Si wafer, the positively charged mobile ions move toward AlO*_x_*(or HGD)/InO*_x_* interface. Then, these positively charged mobile ions accumulate electrons in the InO*_x_* channel, forming an EDL at the AlO*_x_*(or HGD)/InO*_x_* interface. This in turn increases the areal capacitance of the device. The different capacitance with applied bias frequency may constitute solid evidence of the formation of an EDL layer. The lower capacitance with larger hysteresis at high frequency (1 MHz) is likely due to the less responsive mobile ions inside the AlO*_x_* films.

**Figure 3:**
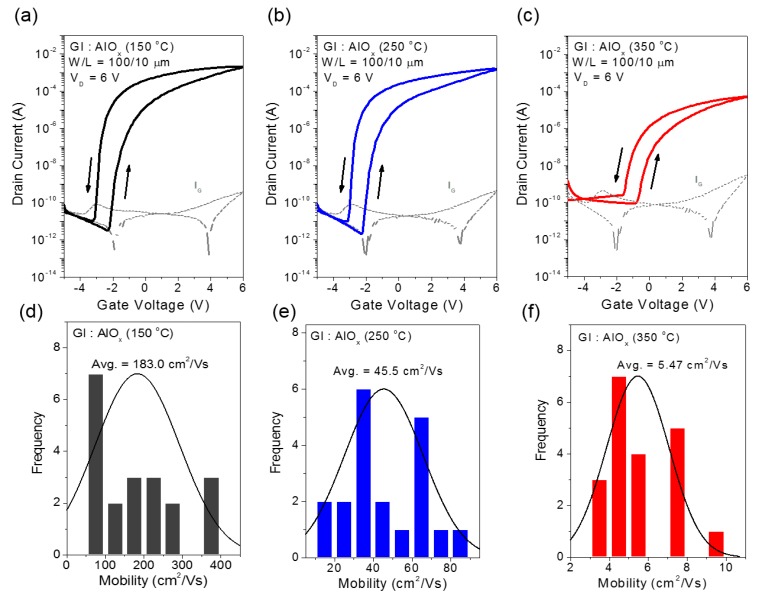
Electrical characteristics of various solution-processed InO*_x_* TFTs on an AlO*_x_* dielectric layer for the different processed conditions. The transfer curves for InO*_x_* TFTs with (**a**) 150 °C-annealed; (**b**) 250 °C-annealed; and (**c**) 350 °C-annealed AlO*_x_* films; the dotted lines indicate the gate leakage current. Statistical distribution of field-effect mobilities for InO*_x_* TFTs with (**d**) 150 °C-annealed; (**e**) 250 °C-annealed; and (**f**) 350 °C-annealed AlO*_x_* films.

Using the solution-processed AlO*_x_* and HGD layers as a gate dielectric, InO*_x_* TFTs were fabricated. Figure 3a–c show the transfer characteristics of InO*_x_* TFTs fabricated with AlO*_x_* gate dielectric layer annealed at different temperatures. In the case of the 150 °C-annealed AlO*_x_* gate dielectric, an average field-effect mobility of 183.0 cm^2^/Vs was observed, which is remarkably high as compared to those made with SiO_2_ gate dielectrics [23,24]. The exceptionally high mobility observed using the AlO*_x_* gate dielectric can be attributed to the formation of an EDL due to a large amount of residual –OHs inside the AlO*_x_* films [25]. In addition to the positive gate bias, a substantial number of electrons are accumulated near the AlO*_x_*/InO*_x_* interface due to the EDL formation. These additional electrons contribute to the drain current resulting in a high drain current and increased field-effect mobility, as well as a large counter-clockwise hysteresis. As mentioned above, the formation of EDL is due to the mobile ions, including –OH residues in the AlO*_x_* gate dielectric layer. As shown in Figure 3b,c, the drain current and the field-effect mobility decreased as the annealing temperature increased. Particularly, with annealing temperatures of 250 °C and 350 °C, the field-effect mobility was decreased to 45.5 cm^2^/Vs and 5.47 cm^2^/Vs, respectively. At a higher annealing temperature, the number of mobile ions dramatically decreases, which drives the formation of a weak EDL and a low carrier concentration at the AlO*_x_*/InO*_x_* interface.

**Figure 4:**
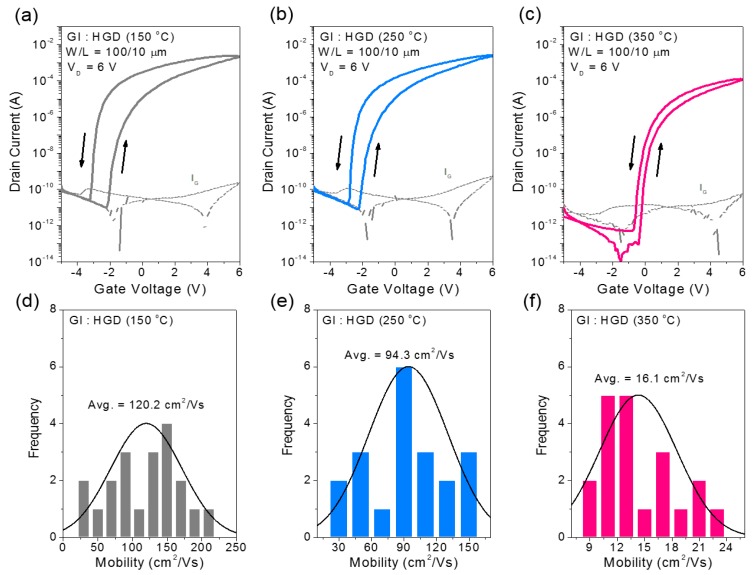
Electrical characteristics of various solution-processed InO*_x_* TFTs on an HGD dielectric layer for the different processed conditions. The transfer curves for InO*_x_* TFTs with (**a**) 150 °C-annealed; (**b**) 250 °C-annealed; and (**c**) 350 °C-annealed HGD films. The dotted lines indicate the gate leakage current. Statistical distribution of field-effect mobilities for InO*_x_* TFTs with (**d**) 150 °C-annealed; (**e**) 250 °C-annealed; and (**f**) 350 °C-annealed HGD films.

In the case of the HGD gate dielectric layer, similar behavior was observed. Figure 4a–c show the transfer characteristics of InO*_x_* TFTs fabricated with the HGD gate dielectric layer annealed at different temperatures. The InO*_x_* TFTs fabricated with a 150 °C-annealed HGD gate dielectric showed an average field-effect mobility of 120.2 cm^2^/Vs. Additionally, in the case of 250 °C- and 350 °C-annealed HGD gate dielectrics, field-effect mobilities of 94.3 cm^2^/Vs and 16.1 cm^2^/Vs were observed, respectively, showing a similar decreasing trend with the annealing temperature. This decreasing trend of mobility with annealing temperature can also be attributed to the decrease of the mobile ions and corresponding weak EDL formation. The relatively superior mobility in the HGD devices can be described by the aforementioned polymer network confinement of the mobile ions in the HGD [20]. Nonetheless, the solution-processed HGD gate dielectrics allow the acceptable formation of EDL at the gate dielectric/InO*_x_* interface, which significantly enhances the electrical properties of InO*_x_* TFTs compared to the bare AlO*_x_* films. In addition, due to the high dielectric constant and low thickness of the gate dielectric layers, low voltage operation below 6 V was possible, enabling low power consuming electronic devices.

**Figure 5:**
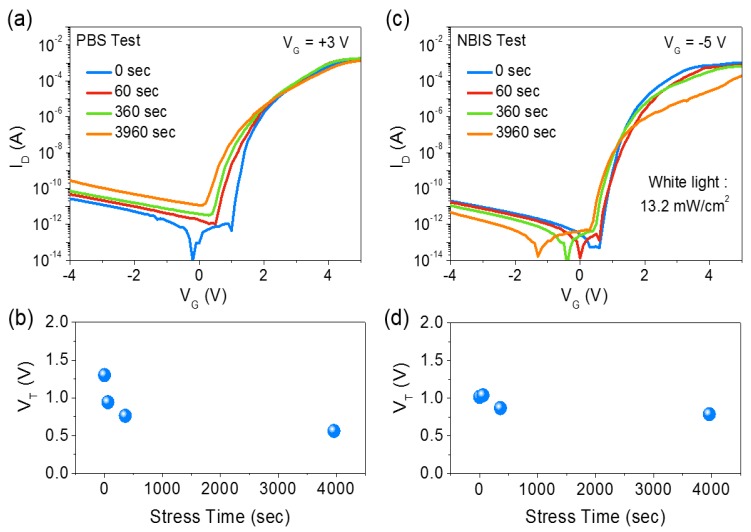
(**a**) Positive gate bias stability and (**b**) evolution of threshold voltage (V_T_) of solution-processed InO*_x_* TFTs with the 150 °C-annealed HGD dielectric layer (V_GS_ = +3 V, t = 3960 s); (**c**) negative gate bias illumination stability and (**d**) evolution of threshold voltage (V_T_) of solution-processed InO*_x_* TFTs with the 150 °C-annealed HGD dielectric layer (V_GS_ = −5 V, t = 3960 s).

**Figure 6:**
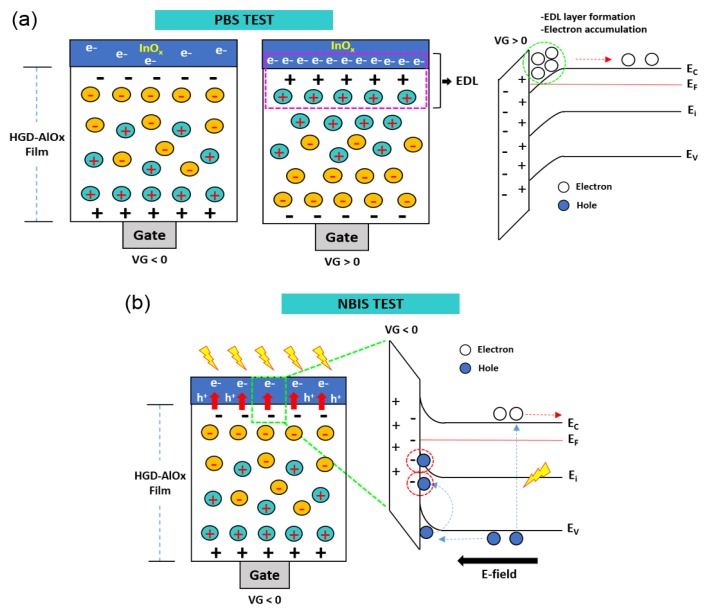
Schematic and energy band diagrams of corresponding V_T_ instability of solution-processed InO*_x_* TFT with the 150 °C-annealed HGD dielectric layer for (**a**) PBS and (**b**) NBIS tests.

In oxide semiconductor-based TFTs, the operational stabilities under positive gate bias stress (PBS) and negative illumination gate bias stress (NBIS) are important factors. In order to determine the operational stability of InO*_x_* TFTs using the HGD gate dielectric, PBS and NBIS tests were carried out. Figure 5a shows the variation of transfer characteristics in InO*_x_* TFTs under PBS. Here, the gate bias was set at +3 V, and the transfer curves were measured at 60, 360, and 3960 s. As shown in Figure 5b, the device exhibited reasonably small threshold voltage shift (ΔV_T_ = −1.67 V) during the 3960 s of PBS. Typically, under PBS conditions, oxide TFTs tend to have positive V_T_ shift due to the electron trapping at the gate dielectric/semiconductor interface [26,27,28]. However, in the case of InO*_x_* TFTs using the HGD gate dielectric, a negative V_T_ shift was observed. This opposite behavior in V_T_ instability is possibly caused by the mobile ions present in the HGD gate dielectric. Particularly, under a continuous PBS condition, a significant amount of positively charged mobile ions move towards the InO*_x_*/HGD interface (Figure 6a). These positive mobile ions, then, contribute to the accumulation of electrons in the InO*_x_* channel layer. However, due to the slow responsive characteristic of mobile ions [29,30,31], they tend to remain near the InO*_x_*/HGD interface causing a negative shift of transfer curves. In addition, the stability under the NBIS condition was also analyzed. Figure 5c,d show the variation of transfer characteristics and V_T_ under NBIS condition, respectively. Surprisingly, the InO*_x_* TFT showed extremely high stability against NBIS and showed ΔV_T_ of −0.23 V (after 3960 s). Typically, the NBIS causes a significant negative V_T_ shift due to the ionization of neutral oxygen vacancies and negative-bias-induced hole injection in the gate dielectric layer [32,33,34,35]. However, the InO*_x_* TFT with HGD gate dielectric showed negligible V_T_ shift under a prolonged light illumination and negative bias condition. This significant reduction in the V_T_ shift can be attributed to the neutralization of injected holes with the negatively charged mobile ions near the InO*_x_*/HGD interface (Figure 6b). As a consequence, the device can exhibit a high operational stability under NBIS. Furthermore, it should be noted that the InO*_x_* TFTs with an HGD dielectric layer exhibited fast recovery after the PBS and NBIS tests. It was found that the V_T_ was recovered to original states after around 6 min, which can be attributed to the gradual neutralization of EDL during the recovery.

## 4. Conclusions

In this paper, we demonstrated high-mobility, operationally stable InO*_x_* TFTs by using ionic-type AlO*_x_* and HGD gate dielectrics. The solution-processed AlO*_x_* and HGD exhibited reasonably high capacitance and dielectric constant, and only a small decrease in capacitance was observed at high frequencies, enabling a high frequency operation. Using the solution-processed HGD gate dielectrics with 350 °C annealing treatment, solution-processed InO*_x_* TFTs were fabricated with an average field-effect mobility of 16.1 cm^2^/Vs with minimal hysteresis. In addition, the HGD gate dielectric also suppressed the negative V_T_ shift during the NBIS condition, which enhanced the operation stability of the InO*_x_* TFTs.
